# Positive parenting practices support children at neurological risk during COVID-19: a call for accessible parenting interventions

**DOI:** 10.3389/fpsyg.2024.1328476

**Published:** 2024-04-08

**Authors:** Rivka Green, Janaksha Linga-Easwaran, Carly Goodman, Marin Taylor, Giulia F. Fabiano, Steven P. Miller, Tricia S. Williams

**Affiliations:** ^1^Department of Psychology, Division of Neurology, The Hospital for Sick Children, Toronto, ON, Canada; ^2^Department of Pediatrics, Faculty of Medicine, BC Children's Hospital, Vancouver, BC, Canada; ^3^Department of Psychiatry, University of Toronto, Toronto, ON, Canada

**Keywords:** positive parenting, family well-being, neurodevelopmental conditions, neurological conditions, mental health

## Abstract

Children and youth with neurological and/or neurodevelopmental conditions were at high risk for behavioral and mental health challenges during the COVID-19 pandemic. Positive and responsive parenting practices may be one way to prevent and manage potential difficulties in families. We aimed to identify whether positive parenting practices were associated with reduced behavioral concerns in children at neurological risk during the late stages and aftermath of the COVID-19 pandemic. In addition, we examined whether ongoing parental stress, anxiety, and depression impacted parenting practices during this time period. Families (*N* = 179) with children 4 to 15 years old (*M* = 7.11y, *SD* = 2.02) diagnosed with neurological (84.3%), neurodevelopmental (54.8%) or comorbid neurological and/or neurodevelopmental conditions (21.2%) were contacted to complete online questionnaires regarding demographics, parent stress, child behavior, COVID-19 conditions, and parenting practices. Multivariable linear regression (MLR) analyses examined the association between positive parenting practices and parenting competency measures with child behavioral outcomes, controlling for relevant covariates, including COVID-19 related stress. MLR were also run to determine whether parental mental health impacted parenting practices. More positive parenting practices predicted fewer child problem behaviors and lower intensity of problem behaviors. Similarly, a higher sense of satisfaction with parenting competence also predicted fewer child problem behaviors and lower intensity of problem behaviors. In addition, higher reported parental depression, anxiety, and stress significantly predicted fewer reported positive parenting practices. Findings points to the promising application of positive parenting interventions to support vulnerable families, as well as the need for parental mental health intervention to support parenting practices.

## Introduction

The COVID-19 pandemic and associated restrictions introduced unprecedented challenges to families’ lives, including to physical and mental health, behavior, friendships, and education, with variability between developmental and sociodemographic groups (Prime et al., 2020; Masten, 2021; Poulain et al., 2022). In general, children at high neurological risk (including those with early brain injuries, genetic conditions, or neurodevelopmental conditions such as Autism Spectrum Disorder and Attention Deficit Hyperactivity Disorder often face additional neurodevelopmental challenges such as higher rates of child behavioral problems and psychological distress (Tak and McCubbin, 2002; Sikora et al., 2013; Bemister et al., 2014; Wiener et al., 2016; Williams et al., 2017). During and following COVID-19 specifically, children and adolescents with pre-existing neurological conditions (i.e., epilepsy, encephalopathy, early brain injury) were at a greater risk of behavioral and mental health deterioration—including but not limited to higher rates of anxiety, depression, and stress due to isolation (Gassman-Pines et al., 2020; Bova et al., 2021; Williams et al., 2021; Zijlmans et al., 2021; Cost et al., 2022). Children with neurodevelopmental disorders showed the same patterns of detrimental effects on mental health due to the pandemic (Conti et al., 2020; Guller et al., 2021; Masi et al., 2021), with as many as 40–50% of Canadian children impacted by emotional and behavioral difficulties, conduct problems and irritability Nonweiler et al., 2020; Cost et al., 2022).

Parents also faced mental health challenges during the COVID-19 pandemic: a meta-analysis involving nearly 9,000 participants revealed that mothers of young children (under the age of 5 years) experienced higher rates of clinically significant depression and anxiety symptoms during the pandemic (Racine et al., 2021). The pooled prevalence rates for depression and anxiety were found to be 26.9 and 41.9%, respectively, indicating an increase compared to pre-pandemic estimates. Parental stress and mental health deterioration was found to negatively impact children’s behavior during the COVID-19 pandemic (Shoychet et al., 2022). In line with the research conducted on the general population, children at neurological risk had greater COVID-19-related mental health deterioration when parents reported more mental health concerns (Williams et al., 2021).

In addition to the children themselves, parents of children with behavioral and mental health challenges may also have difficulties coping with additional stressors (Compas et al., 2017), including those further exacerbated by the pandemic (Theberath et al., 2022). Prime et al. (2020) introduced the COVID-19 disruption model, suggesting that pandemic-related social disruptions negatively influence parental well-being, which subsequently impacts various aspects of family functioning. The identification of factors that reduce behavioral challenges and promote optimal function in this population is needed to inform appropriate interventions.

One factor that may positively impact children’s mental health in at-risk families is positive parenting (Black and Lobo, 2008; Torres Fernandez et al., 2013; Daks et al., 2020; Guruge et al., 2021). Positive parenting includes parental flexibility, parental responsiveness, constructive parenting, and emotional warmth (Tamis-Lemonda et al., 1996), and it can lead to more positive parent–child relationships – a well-established determinant of well-being in children (von Suchodoletz et al., 2011; Seay et al., 2014; Weeland et al., 2021; Koper et al., 2022; Shoychet et al., 2022; Wang et al., 2022; Yates and Mantler, 2023). For example, positive parenting practices with children with ADHD can act as a protective factor against conduct problems (Chronis et al., 2007; Healey et al., 2011; Dvorsky and Langberg, 2016). The extent to which these positive parenting factors were associated with optimal behavioral outcomes in children at neurological risk during the COVID-19 pandemic remains largely unexplored.

One COVID-19 specific study found that teaching parents how to engage with their children through acts of kindness, developing trusting relationships, and responding with compassion increased parent–child resilience during the pandemic as measured by parental resilience and parent-reported child empathetic prosocial behavior levels (Johnson et al., 2022). Studies of children with neurodevelopmental disorders, such as autism spectrum disorder and intellectual disabilities, showed that positive parenting enhanced pandemic resilience, as measured by a family quality of life and parent–child relationship questionnaires (Bolbocean et al., 2022). However, no studies have looked at whether positive parenting can specifically impact children’s behavior in a sample of children at neurological risk (i.e., preterm birth, brain injuries, pediatric stroke) and/or neurodevelopmental (i.e., learning disabilities, autism, ADHD) during and immediately following the COVID-19 pandemic, and whether parental mental health impedes positive parenting practices.

Identifying potential protective factors, such as parenting practices, that promote positive behavioral outcomes during times of increased stress are essential for informing targeted interventions for vulnerable families. In addition, it is important to consider factors that contribute to or impede effective parenting practices. Therefore, this study aimed to examine (1) whether self-reported positive parenting practices were associated with fewer child behavioral challenges during the COVID-19 pandemic amongst an at-risk neurological and/or neurodevelopmental Canadian sample. Secondly, we examined (2) whether self-reported parental mental health was associated with parenting practices.

## Methods

### Research design

Participants were recruited from three larger studies if they had consented to future follow-up for research: the first study (REB #1000054921) was an observational study of parents of children with neonatal brain injuries to examine factors related to parent stress and well-being. The other two studies (REB #1000072960 and #1000063660) were pilot studies for a virtual positive parenting intervention targeting child behavior and family well-being (I-InTERACT-North). All three studies were reviewed and approved by the Research Ethics Board at The Hospital for Sick Children. Recruitment and data collection took place between March 2022 and January 2023.

Consenting parents were forwarded online questionnaires via REDCap (Harris et al., 2009), a secure web application housed on a secure server.

### Participants

Recruitment and data collection took place between March 2022 and January 2023. Participants were parents of children with neurological and/or neurodevelopmental conditions who were followed at The Hospital for Sick Children or part of the Province of Ontario Neurodevelopmental Disorders Network (POND).

Neurological conditions included those related to early brain injury and associated medical conditions, such as stroke, hypoxic ischemic encephalopathy (HIE), prematurity, epilepsy, and congenital heart disease (CHD). Neurodevelopmental conditions included attention deficit hyperactivity disorder (ADHD) and autism spectrum disorder (ASD). Patients with neurological conditions often had co-occuring neurodevelopmental conditions. Consenting parents were forwarded online questionnaires via REDCap (Harris et al., 2009), a secure web application housed on a secure server. Exclusion criteria from prior studies included child age of less than 3 years or major medical issues requiring ongoing inpatient care (e.g., significant surgical or inpatient treatment).

A total of 305 families were invited to complete the follow-up questionnaires between March and October 2022. Families (*N* = 179) who completed the surveys during this data collection period were included in analyses. The final sample consisted of predominantly English-speaking, European, college/university-educated, and two-parent households. The children in these families were between 4 and 15 years old (*M* = 7.11y, SD = 2.02y; see Table 1) at the time of questionnaire completion. Thirty-eight children (21.2%) had comorbid neurological and/or neurodevelopmental conditions; both stroke (24.0%) and ADHD (24.6%) affected one-quarter of this population.

**Table 1:** Family demographics and child clinical characteristics.

		Family characteristics (*N* = 179)
Parent 1* relationship to child, n (%)		
	Mother	150(83.8%)
	Father	9(5.0%)
	Other^a^	19(10.6%)
*Parent 1 Reported Heritage Culture* *^c^* *, n (%)*		
	African	4(2.2%)
	Asian (East, South, South-East)	31(17.3%)
	European	90(50.3%)
	Indigenous (First Nations, Inuit, Metis)	4(2.2%)
	Hispanic or Latino	8(4.5%)
	Middle Eastern	7(3.9%)
	Prefer not to say/no response	28(15.6%)
*Parent 1 Education* *^c^* *, n (%)*		
	High school diploma or less	21(11.7%)
	College/University degree or higher	152 (84.9%)
*Marital Status of Parents, n (%)*		
	Married/Common Law	152(84.9%)
	Separated/Divorced	13(7.3%)
	Other/Would rather not say	12(6.7%)
*Number of siblings living with child, n (%)*		
	None	32(17.9%)
	One sibling	96(53.6%)
	Two or more siblings	49(27.3%)
English as Primary Language – *n* (%)		149(83.2%)
Age of Child M(SD)		7.11y(2.02y)
Gender of Child – Male, n (%)		108(60.3%)
*Child Conditions, n (%)* *^e^*		
	ADHD	44(24.6%)
	Autism Spectrum Disorder	30(16.8%)
	Intellectual Disability and/or Global Developmental Delay	11(6.1%)
	Anxiety and/or Obsessive Compulsive Disorder	11(6.1%)
	Epilepsy	14(7.8%)
	Congenital Heart Defect	34(19.0%)
	Stroke	43(24.0%)
	Cerebral Palsy	14(7.8%)
	Hypoxic Ischemic Encephalopathy	33(18.4%)
	Premature	6(3.4%)
	Genetic Condition	7(3.9%)
	Other Neurodevelopmental Disorders^f^	13(7.3%)
Child Behavior Score, *M(SD)*		
	ECBI Intensity T Score	53.98(9.43)
	ECBI Problem T Score	54.72(11.46)

*Parent 1 refers to parent completing the questionnaire.

a Unspecified relationship (*n* = 17), grandparent (*n* = 2).

b grandparent (*n* = 2), stepfather (n = 7).

c Counts reflect the total number of endorsed cultures/heritages within and across participants, as many parents provided more than one classification (e.g., bi-cultural influences). ^d^Education and employment status were based on primary income earner.

e Other languages included Arabic, Armenian, Assyrian, Cantonese, Cayuga, Creole, Dari, Farsi, French, Hindi, Indonesian, Italian, Ukrainian, Ojibiwe, Pashto, Polish, Portuguese, Spanish, Tagalog, Urdu, and Yoruba.

f Counts reflect the total number of endorsed conditions for a child, as many have multiple diagnoses.

g Other: Learning disability (*n* = 7), oppositional defiant disorder (*n* = 3), sensory processing disorder (*n* = 3).

### Measures

#### Demographics questionnaire

A comprehensive questionnaire was used to collect child and parent demographic information and child clinical information. Race and cultural heritage data was reported according to updated categories proposed by Canadian Census data and the Canadian Institutes of Health Research (Canadian Institute for Health Information, 2022). To accommodate for those of multi-ethnic origins or identities, the total number of responses were greater than the total number of participants (Statistics Canada, 2021). Neurological and neurodevelopmental-relevant medical data were collected through medical chart reviews, where available, and through parent report of child diagnostic history (open ended text box).

#### COVID-19 questionnaires

A subset of questions from the CoRonavIruS Health Impact Survey (CRISIS) V0.1 Parent/Caregiver Baseline Form and the CRISIS Adult-Self Report Baseline Form (Nikolaidis et al., 2021) were included in the background questionnaire to assess social and financial impacts of the pandemic on families. The CRISIS questionnaire, designed by an international collaboration, examines mental health during the COVID-19 pandemic for families of children with neurodevelopmental conditions and has been used in larger COVID-19 studies (Hawke et al., 2020; Nikolaidis et al., 2021; Cost et al., 2022). The original Parent/Caregiver baseline survey includes 98 questions regarding child wellbeing and mental health, and the original Adult-Self Report baseline survey includes 99 questions about parent wellbeing and mental health during COVID-19 and the impact of specific stressors (e.g., social isolation, COVID-19 economic concern, changes in relationships and positive changes related to the pandemic). The survey was adapted and abbreviated to 14 questions regarding the parent and 11 for the child based on feedback from our family advisory team in effort to reduce participant burden (e.g., reduce time to complete questionnaire and redundancy in questions). A 7-item subscale of the COVID Family Stressor Scale (Prime et al., 2021) asked parents to report on COVID-19-related family stress and changes in the home (α = 0.82). Each item is rated on a 3-point scale of not true, somewhat true, and very true where higher sum scores indicate greater family stress related to COVID-19 changes in the home.

#### Parent sense of competence

The Parenting Sense of Competence Scale (PSOC; Johnston and Mash, 1989), a 17-item parent-reported survey measuring parents’ sense of confidence in their ability to parent children aged 2–9 years old, was used to assess self-reported parenting competence. Sample statements include “I meet my own personal expectations for expertise in caring for my child” and “Being a parent makes me tense and anxious,” each rated on a six-point scale ranging from 1 (strongly disagree) to 6 (strongly agree). An efficacy score is calculated using 9 items, and a satisfaction score is calculated using 7 items. A higher score indicates a greater sense of parenting efficacy or satisfaction. The PSOC has appropriate test–retest reliability, ranging between 0.73–0.74 and internal consistency (α = 0.80; Ohan et al., 2000).

#### Parenting practices

An abbreviated version of the 2014 Ontario Child Health Study’s Parent Practices Scale (PPS; Boyle et al., 2019) was used to assess frequency of positive and negative parenting practices. The modified PPS consists of 12 parent-report questions with Likert-item response options ranging from 1 (not at all) to 5 (more than 10 times) and asks parents to report the frequency of various parenting practices over the past six months. This scale specifically focuses on parenting behaviors, rather than beliefs, and asks questions pertaining to aspects of parental support and engagement (i.e., listening to child’s ideas, responsivity, presence), as well as hostility and coercion (i.e., threatening punishment). A higher score indicates greater positive parenting. The PPS has appropriate test–retest reliability (α = 0.81; Prime et al., 2021).

#### Child behavior

The Eyberg Child Behavior Inventory (ECBI; Eyberg and Pincus, 1999) was used to assess child behavior concerns. The ECBI is a 36-item parent-reported measure that assesses the frequency and severity of a child’s current problematic behavior in the home on a seven-point intensity scale and yes/no problem scale. The ECBI yields a total problems T score (“Is this [behavior] a problem for you?”; clinical cut-off T-score = 60) and a total intensity T score (“How often does this [behavior] occur?”; clinical cut-off T-score = 60). The ECBI has good test–retest reliability, ranging from 0.75 to 0.86, appropriate internal consistency (α = 0.94), and is sensitive to effects of family treatments (Funderburk et al., 2003).

#### Parent mental health

The Depression and Anxiety Stress Scale (DASS; Lovibond and Lovibond, 1995; Antony et al., 1998) was used to assess self-reported symptoms of parent stress, depression, and anxiety. The DASS consists of 42 items that are equally divided among three different scales: depression (α = 0.97), anxiety (α = 0.92), and stress (α = 0.95). Each item is rated on a scale from zero (never) to three (almost always) where higher scores indicate more severe symptoms of depression, anxiety, or daily stress. On the DASS-21, a summary score ≥ 13, ≥ 9, or ≥ 18 indicates elevated parenting depression, anxiety, or stress, respectively.

#### Analyses

Demographic variables were analyzed using cross tabulations, means and standard deviations. All relationships between variables of interest, including predictors, outcomes, and covariates, were analyzed using a series of correlations. Multivariable linear regression analyses were run using parenting competence and practice measures as predictors and child behavior and child mental health as dependent variables. Possible covariates included stress due to COVID, parent age, child age, parent culture, gender, marital status, number of siblings, birth order, parent education, whether the child had a medical diagnosis, and whether the child had a neurodevelopmental disorder. Covariates were retained in the model only if they had a p-score equal to or less than 0.2 and/or changed the predictor beta coefficient by more than 10% when included. Final covariates are described in the results. We also considered possible interaction terms by COVID-related family stress and parental mental health, which may influence the relationship between parenting and child behavior. We controlled for multiple comparisons using the Bonferroni method by taking the number of tests and dividing by the alpha value. In addition, we examined whether parental mental health predicted parenting practices by regressing PPS by parental stress, depression and anxiety, controlling for covariates, including COVID-stress.

## Results

### Participants profile

Overall, child behavioral scores were slightly elevated, and mean parental mental health scores were within average score ranges; however, there was wide variability in scores.

The most common child condition reported in our sample was ADHD, followed by acquired brain injuries such as stroke, HIE, and CHD; less frequently reported conditions in our sample included epilepsy, ASD, and global developmental delay (Table 1). Many families reported multiple diagnoses, most commonly ADHD, along with a neurological condition, such as stroke (Table 1).

### COVID-19 descriptives

Within our study sample, the majority of parents retained their jobs during the pandemic and continued with 8% reporting experiencing financial difficulties and 6% reporting feeling ‘very’ or ‘extremely’ worried due to their living situation. About 30% of families reported receiving funds from COVID-19 government relief programs. The majority of families (61%) reported slight to moderate levels of stress when it came to taking care of their child, their education and daily activities during COVID, and 24% reported extreme levels of stress. However, 47% of families still reported that COVID had a positive change in their lives.

### Positive parenting practices, child behavior, and mental health

Overall, child behavioral scores were slightly elevated (Table 1) and mean parental mental health scores were within average score ranges (Table 2); however, there was wide variability in scores. The majority of behavioral problems endorsed included non-compliance (i.e., refusing to listen), exhibiting temper tantrums (including yelling), and difficulty concentrating and finishing tasks. On open-ended responses on questionnaires parents most frequently endorsed their children’s challenges with emotional regulation (i.e., tantrums, meltdowns) and organizational skills (i.e., task planning, taking initiative).

**Table 2:** Predictors of child behavior problems and intensity during COVID-19 in neurologically at-risk children.

Predictor^a^	Outcome	Coefficient (B)	Adjusted *r*^2^	*p*-value
Positive parenting practices	Behavior problems	−0.889	0.325	**<0.001**
	Behavior intensity	−0.843	0.338	**<0.001**
Parenting sense of competency^b^ – satisfaction	Behavior problems	−0.425	0.220	**<0.001**
	Behavior intensity	−0.477	0.238	**<0.001**
Parenting Sense of Competency – Efficacy	Behavior problems	−0.182	0.162	0.164
	Behavior intensity	−0.136	0.119	0.218

a Final covariates included child age at consent, gender, diagnosis of a neurodevelopmental or mental health disorder, COVID-19 related stress, parents’ marital status, and European versus other.

b Given that the Parenting Sense of Competence Scale has been validated only for children aged 2–9 years, we repeated these analyses excluding children aged 10–15 (*n* = 24). The results did not change.

**Table 4:** Parental depression, anxiety, and stress predicts positive parenting practices.

Predictor^a^	*M* (*SD*)	Outcome	Coefficient (*B*)	Adjusted *r*^2^	*p*-value
DASS – Depression	9.99 (8.2)	Positive parenting	−0.40	0.27	**<0.001**
DASS – Anxiety	4.27 (5.51)	Positive parenting	- 0.51	0.22	**<0.001**
DASS – Stress	6.28 (8.11)	Positive parenting	−0.42	0.25	**<0.001**

a Final covariates included child age at consent, gender, diagnosis of a neurodevelopmental or mental health disorder, COVID-19 related stress, parents’ marital status, and European versus other.

Parents rated themselves as having moderate to good parenting practices (PPS: *M* = 40.68/53, *SD* = 5.78); however, there was a large range of scores from very low (minimum: 21) up to a perfect score (maximum = 53). Higher reported positive parenting practices were strongly correlated with lower child behavior intensity (*r* = − 0.55), and moderately correlated with fewer behavioral problems (*r* = − 0.49). A moderate positive correlation was found between positive parenting and parenting competency [both efficacy (*r* = 0.41) and satisfaction (*r* = 0.43)]. Greater positive parenting practices had small to moderate inverse correlations with scores on parental depression (*r* = − 0.30), anxiety (*r* = − 0.17), and stress (*r* = − 0.40).

### Covariates

Child age, child gender, number of siblings in the home, and parent level of education had negligible associations with positive parenting practices, parental mental health, and parenting competency (*r* < ± 0.1). However, parental mental health had low to moderate correlations with reported financial strains during the pandemic (*r* = 0.24–0.37), and therefore we retained this variable for regressions. Additionally, COVID-related family stress was inversely and moderately correlated with parenting competency, positive parenting, child behavioral problems, and parental mental health (*r* < − 0.24); therefore, it was also retained in analyses to determine whether parenting practices and/or parental mental health remained predictors after accounting for COVID-family stress. Parental mental health and COVID-related stress were not significant effect modifiers in regression analyses, both as continuous variables and dichotomized by clinical cut-offs.

### Predicting child behavior

After controlling for covariates, higher positive parenting practices predicted fewer child problem behaviors (*B* = −0.889, *p* < 0.001) and lower intensity of problem behaviors (B = −0.843, *p* < 0.001; Table 3). Similarly, a higher sense of satisfaction with parenting competence predicted fewer child problem behaviors (B = –0.425, *p* < 0.001) and lower intensity of problem behaviors (*B* = −0.477, *p* = <0.001). A higher sense of parental competency was not significantly associated with behavioral outcomes.

**Table 3:** Predictors of child behavior problems and intensity during COVID-19 in neurologically at-risk children.

Predictor^a^	Outcome	Coefficient (B)	Adjusted *r*^2^	*p*-value
Positive parenting practices	Behavior problems	−0.889	0.325	**<0.001**
	Behavior intensity	−0.843	0.338	**<0.001**
Parenting sense of competency^b^ – satisfaction	Behavior problems	−0.425	0.220	**<0.001**
	Behavior intensity	−0.477	0.238	**<0.001**
Parenting sense of competency – efficacy	Behavior problems	−0.182	0.162	0.164
	Behavior intensity	−0.136	0.119	0.218

a Final covariates included child age at consent, gender, diagnosis of a neurodevelopmental or mental health disorder, COVID-19 related stress, parents’ marital status, and European versus other.

b Given that the Parenting Sense of Competence Scale has been validated only for children aged 2–9 years, we repeated these analyses excluding children aged 10–15 (*n* = 24). The results did not change.

### Predicting positive parenting

After controlling for covariates, higher reported parental depression (*B* = −0.40, *p* < 0.001), parental anxiety (*B* = −0.51, *p* < 0.001), and parental stress (*B* = −0.42, *p* < 0.01) significantly predicted fewer reported positive parenting practices (Table 4).

## Discussion

The COVID-19 pandemic and its aftermath was a very stressful period for Ontario families. School closures, loss of social, therapeutic, and recreational activities, as well as loss and modifications to work environments led to significant changes in the lives of children and parents. Children with pre-existing conditions were at elevated risk for behavioral and mental health challenges during the pandemic (Cost et al., 2022), and studies have demonstrated the scope of children affected, which include neurological and neurodevelopmental populations (Williams et al., 2021). In the present study, we aimed to explore positive parenting as a potential mitigating factor for behavioral challenges faced by families of children with neurological and/or neurodevelopmental disorders during the later stages of the COVID-19 pandemic and its aftermath.

We found that self-reported positive parenting practices and reported parenting competence were predictive of lower child behavior problems and reduced intensity of those problems. Specifically, the more positive parenting practices endorsed (i.e., listening to child, responding to child) was associated with fewer behavioral concerns (i.e., less yelling, fewer tantrums, following directions), as well as a lower intensity of these problems (i.e., less severe tantrums and melt downs). As well, a higher degree of parenting competence (i.e., meeting one’s own parenting expectations, feeling less tense in parenting) was also associated with fewer behavioral problems and a lower intensity of these problems mentioned above. In addition, we found that poorer reported parental mental health (i.e., more symptoms of depression, anxiety, stress) was associated with fewer positive parenting practices (i.e., threatening punishment, less responsivity).

Overall, our findings are consistent with previous studies of children with ADHD and non-clinical samples, where parenting stress negatively impacted parenting style, and was related to child impairment, externalizing behaviors and poorer overall functioning (Cina et al., 2011; Healey et al., 2011; Flannery et al., 2021; Okorn et al., 2022). While other studies have demonstrated the relationship between positive parenting, behavior, and parental mental health, our study included children and families with *pre-existing* neurological and neurodevelopmental conditions during the COVID-19 pandemic, who have been shown to be specifically at a high risk for behavioral and mental health challenges generally, and even more so during the pandemic (Conti et al., 2020; Guller et al., 2021; Masi et al., 2021). Therefore, identifying protective factors to mitigate these challenges is even more important in these populations.

We found that positive parenting skills may serve as a modifiable target for child and family mental health interventions in order to mitigate declines in mental health and behavior when unforeseen circumstances (i.e., COVID-19 pandemic) occur. Our study demonstrated that the relationship between positive parenting and behavior remained strong even after considering factors such as, parental level of education and COVID-19 related family stressors, highlights the potential of positive parenting and family wellness to be able to supersede even the crises and lack of services during the pandemic. Considering that some studies have shown that level of parental education impacts aspects of positive parenting (Planalp et al., 2022), it is important to design interventions that can be accessible and relevant to all families.

In considering the limitations of the current study, it is important to note that all data used in this study to describe child behavior, child mental health, parenting skills, and parenting competency were collected at one time period and focused solely on parent report data. A previous study on parent affect during COVID-19 leveraged the child’s perspective by recruiting older children (*M* = 10.5y; Chu et al., 2021) to complete modified questionnaires as a means of engaging multi-informants. Similar efforts to understand children’s experiences may be helpful in future studies. Given that this is a cross-sectional design, future research may benefit from longitudinal approaches to assess whether parental stress and mental health, as well as child behavior, precede parenting practices or vice versa. Future research on associations with parenting competence would also benefit from a validated scale for children older than 9 years; however, in the present study, we repeated analyses using the PSOC scale while excluding children aged 10–15 years, and results did not change. It is possible that parents of children with fewer behavioral challenges indicate more positive parenting due to fewer challenges in their children’s behavior. Future studies may wish to consider parenting and behavioral associations amongst different age groups and whether there are differences within age-specific stratifications.

In addition, in our study, families tended to consist of higher socioeconomic status and older age, consistent with the demographic of research cohorts (Henrich et al., 2010). Participants in our sample had also participated in previous research studies on parent mental health and well-being, which may have inflicted bias on their responses if they had an idea of the research objectives. Previous research has shown that these qualities tend to exist in families with better social support (Smith et al., 2010); future research may wish to consider a more socioeconomically diverse sample and other diversities not represented in our sample.

### Future implications and clinical considerations

In the unpredictable post-pandemic era, when many environmental factors, such as sociodemographic considerations and previous neurological morbidity, are largely out of parents’ control, the results of this study point to positive parenting as an intervening factor that may have the potential to improve child behavior. In fact, healthcare providers are calling for early interventions to encourage adaptive family relationships (Garner and Yogman, 2021; McCrae et al., 2021). Interventions that help parents cultivate positive relationships with their children, respond to their children’s behavior, and protect their own mental health during times of stress may increase positive parenting and parents’ sense of competence in responding to behavioral concerns. One such example that is applicable to families with medical and neurological backgrounds is the I-InTERACT-North program, which we co-designed to provide virtual, incremental, and individualized 1:1 therapy and coaching for parents of children with neurological challenges (Deotto et al., 2023). I-InTERACT-North was developed as a virtual intervention prior to the COVID-19 pandemic (Burek et al., 2020), and was delivered continuously throughout (Deotto et al., 2023). The results of the present study support emerging research showing that increasing positive parenting is associated with a lower frequency and intensity of child behavioral problems, specifically in vulnerable populations. It is imperative that programs, such as the I-InTERACT-North program, reach families of all backgrounds. Investments in the early years to reduce problematic outcomes must be a priority, and must be accessible to all families, especially those that may be less likely to access these supports (Garner and Yogman, 2021; McCrae et al., 2021). Specifically, parents of children with medical needs have indicated they want support after receiving information on neurodevelopmental and psychiatric vulnerability (Perlman et al., 2023). Indeed, the World Health Organization guidelines call for increased availability of parenting interventions to prevent poor treatment and enhance parent–child relationships World Health Organization (2022). Decades of research have shown that identifying and intervening as early as possible to prevent the occurrence or worsening of mental health sequelae in childhood is the best way to offset mental health disorders in adulthood. Given global threats and complex emergencies, and considering that pandemic-related risks may disproportionately hit children and families who are already disadvantaged (Fegert et al., 2020), we must be better prepared to address the major mental health challenges of the next several decades, and one way is through offering early interventions to promote resilience. Integrating early interventions through clinical care could optimize parenting and strive to prevent the onset of mental health sequelae.

Parenting can be challenging, especially for children with unique needs. Parenting was exacerbated by the pandemic, with the lack of social support and the heightened state of fear and anxiety interfering with routine and practice. In our sample of neurologically and neurodevelopmentally diverse Canadian children, positive parenting helped to reduce challenging behaviors. It is our hope that positive parenting interventions can be accessed by all families and be leveraged to promote optimal mental health and well-being.

## Data availability statement

The raw data supporting the conclusions of this article will be made available by the authors, without undue reservation.

## Ethics statement

The studies involving humans were approved by the Hospital for Sick Children Research Ethics Board. The studies were conducted in accordance with the local legislation and institutional requirements. The participants provided their written informed consent to participate in this study.

## Author contributions

RG: Conceptualization, Formal analysis, Methodology, Writing – original draft, Writing – review & editing. JL-E: Data curation, Formal analysis, Project administration, Writing – original draft, Writing – review & editing. CG: Formal analysis, Writing – original draft, Writing – review & editing. MT: Writing – original draft, Writing – review & editing, Formal analysis. GF: Data curation, Project administration, Writing – original draft, Writing – review & editing. SM: Supervision, Writing – original draft, Writing – review & editing. TW: Conceptualization, Methodology, Supervision, Writing – original draft, Writing – review & editing.
